# Intra-Individual Double Burden of Malnutrition among Adults in China: Evidence from the China Health and Nutrition Survey 2015

**DOI:** 10.3390/nu12092811

**Published:** 2020-09-14

**Authors:** Qiumin Huang, Liusen Wang, Hongru Jiang, Huijun Wang, Bing Zhang, Jiguo Zhang, Xiaofang Jia, Zhihong Wang

**Affiliations:** National Institute for Nutrition and Health, Chinese Center for Disease Control and Prevention, 29 Nanwei Road, Beijing 100050, China; qmhuangx@163.com (Q.H.); wangls@ninh.chinacdc.cn (L.W.); jianghr@ninh.chinacdc.cn (H.J.); wanghj@ninh.chinacdc.cn (H.W.); zzhangb327@aliyun.com (B.Z.); zhangjg@ninh.chinacdc.cn (J.Z.); jiaxf@ninh.chinacdc.cn (X.J.)

**Keywords:** malnutrition, double burden, micronutrient inadequacies, BMI, adults, China

## Abstract

Few studies have focused on quantifying the double burden of malnutrition (DBM) phenomenon in China. We aimed to clarify the prevalence of DBM among Chinese adults as well as to examine whether daily dietary micronutrient status varies by body mass index (BMI) categories. In this study, a sample of 6602 adults aged 18–59 years from the China Health and Nutrition Survey (CHNS) was analyzed. Information was obtained on dietary intake and anthropometric measurements. Dietary intakes of 11 micronutrients were estimated based on the data collected by three consecutive days of 24 h recalls combined with the weighing of household seasonings. Dietary micronutrient deficiency was defined according to the cutoff of the Chinese estimated average requirement (EARs). 44% of Chinese adults faced the problem of DBM, of which nearly 40% experienced overweight/obesity and micronutrient deficiency simultaneously. Comparable percentages (>50%) of Chinese adults had dietary intake less than the Chinese EARs for key micronutrients including retinol, thiamin, riboflavin, vitamin C, calcium, selenium, zinc, and magnesium, and the percentages varied by body weight status. More than 80% participants had at least two selected vitamin or mineral deficiencies in all BMI categories. These findings indicate that Chinese adults have a high DBM and micronutrient inadequacies prevail among and within gender and all BMI categories. All body weight groups need advice on the changing needs for dietary variety to ensure optimal health.

## 1. Introduction

Most countries, at all levels of development, have been slow to acknowledge the challenge of multiple forms of malnutrition, particularly in some developing counties facing the double burden of malnutrition (DBM), which is the coexistence of undernutrition (i.e., underweight and micronutrient deficiencies) along with overweight and obesity, or diet-related noncommunicable diseases, within individuals, households, and populations, and across the lifecourse [1]. Several studies have shown associations between low dietary intake of micronutrients, including vitamins A and C, calcium, magnesium, and zinc, and obesity and body mass index (BMI). In 2012, 33.8% of American adults aged 40–59 years were classified as obese, and the dietary intakes of vitamins A and C, iron, and magnesium were relatively low in obese men and women compared with other BMI categories [2]. In 2014–2016, among England volunteers aged 18–25 years, weight status was significantly associated with micronutrient intake, and a trend towards a decrease in vitamin and mineral intake with increasing weight was noted [3]. Among Chinese studies, previous research on dietary intakes of micronutrients focused on differences in intakes or status by sex or age groups for which the Chinese Dietary Reference Intakes 2013 have been established [4], and previous results reported that women had lower dietary intakes for retinol, vitamins A and C, thiamine, riboflavin, calcium, phosphorus, magnesium, iron, zinc, copper, and selenium compared with men [5,6,7], and a significant higher percentage of women were not meeting the estimated average requirement (EARs) for several nutrients such as retinol, riboflavin, and thiamine without considering their BMI status [5].

To date, we have little understanding of the DBM at intra-individual level, with underweight/overweight/obesity and micronutrient deficiency being present in the same individual, or of micronutrient intakes status by body status among Chinese adults, particularly data in adults by sex are sparse. Therefore, explorations are needed to address this limit and to clarify whether daily dietary micronutrient status varies by BMI categories. In the present article, we focused on quantifying the prevalence and distribution of the DBM among Chinese adults and estimating the dietary micronutrient intakes and the prevalence of nutritional inadequacies among them, using data from the China Health and Nutrition Survey (CHNS, 2015), to further identify differences in micronutrient status from food between BMI categories. Such findings are important for policymakers to support actions to implement interventions to improve diet quality to address undernutrition and overnutrition in Chinese individuals.

## 2. Methods

### 2.1. Study Population 

The current study utilized data from the CHNS, which is a longitudinal household survey with the goal of assessing the relationships between the economic, sociological, and demographic transformation in China. The CHNS was conducted in 9 rounds across 9 provinces during the period of 1989–2009. The 9th round was conducted in 2011 across 12 provinces, and the 10th round was conducted in 2015 across 15 provinces. It used a multistage, stratified, random cluster process to select communities for the sample. The survey background, aims, design, and methods have been reported in detail elsewhere [8].

Our analysis used the 10th-round survey of CHNS in 2015. Of 8213 eligible participants aged 18–59 who had complete data on dietary, anthropometric, demographic, socioeconomic, and other lifestyle factors in a survey year, we excluded those with implausible energy intakes (n = 170; for men consuming <1000 or >6000 kilocalories per day (kcal/d), for women consuming <800 or >5000 kcal/d); pregnant or lactating women (n = 1441). Our final sample was 6602 (3699 men and 2903 women). All participants provided written informed consent, and the Institutional Review Committees of the University of North Carolina at Chapel Hill and the Chinese Center for Disease Control and Prevention approved the survey protocol (201524).

### 2.2. Assessments of Dietary Micronutrient Intakes

Daily dietary intake information was assessed using three consecutive 24 h recalls for each individual and weighed seasonings in the household inventory over the same period in the CHNS. Trained health workers interviewed the participants on each of those days to collect all food consumption (type, amounts, type of meal, and place of consumption) at home and away from home during the preceding 24 h. For the food inventory, all available purchased and stored foods in the household were measured. Changes in household food inventory as well as wastage were used to estimate total household food consumption. We determined the percentage of the oil, salt, and other condiments from the household inventory that each member consumed by the ratio of their energy intake to the energy intake of all members. Based on the dietary intake of each ingredient consumed during the three days, we used the China Food Composition Table to estimate the three-day average intakes of total energy and micronutrients. Retinol was derived from the simplified formula: μgRAE = μg retinol + μg carotenes/12. The detailed diet data have been described elsewhere [9]. Intakes of micronutrients were calculated as absolute intake and as intake/1000 kcal in the analyses.

### 2.3. Anthropometric Measurements

Trained health workers or nurses measured height, weight, and blood pressure following standardized procedures. Weight and height were measured to the nearest 0.1 kg and 0.1 cm, respectively, with the participants in lightweight clothing and without shoes according to validated anthropometry manual standards [8]. Body mass index (BMI) was calculated as body weight in kilograms divided by the square of height in meters (kg/m^2^). A BMI greater than or equal to 30 kg/m^2^ was identified as obese, between 25.0 and 29.9 kg/m^2^ as overweight, between 18.5 and 24.9 kg/m^2^ as normal, and a BMI smaller than 18.5 kg/m^2^ was considered underweight. Blood pressure was measured at least three times using a standard mercury sphygmomanometer after the participants rested at least five minutes in a seated position. Three measurements were obtained with a 30 s interval. Systolic blood pressures (SBPs) were determined at the first appearance of a pulse sound (Korotkoff phase 1), and diastolic blood pressures (DBPs) at the disappearance of the pulse sound (Korotkoff phase 5). We used the mean of three satisfactory measurements for analyses.

### 2.4. Assessment of Covariates

Trained interviewers used standard questionnaires to collect information on demographic and other possible confounding factors, including age, per capita annual family income, education (illiterate, primary school, and ≥high school), smoking (current smokers vs. former or never smokers), alcohol drinking (current drinkers vs. former or never drinkers), physical activity, community urbanization index (score), medical history, and medication use. Physical activity was assessed by a CHNS questionnaire [10,11,12]: participants were asked to report all activities (including four domains: occupational, household chore, leisure time, and transportation activities.) in average hours per week, and we converted the intensity of physical activity assignments into a metabolic equivalent of task (MET) hours per week based on the American College of Sports Medicine Association’s recommended standard [13]. The standardized, validated urbanization measure captures changes in the following 12 dimensions at the community level: population density, economic activity, traditional markets, modern markets, transportation infrastructure, sanitation, communications, housing, education, diversity, health infrastructure, and social services. Each is based on numerous measures applicable to each dimension [14]. The yearly income, physical activity, and urbanicity index were categorized into tertiles (low, medium, and high) in the analyses. Participants with the history of diet-related chronic diseases were defined as having hospital records of stroke, myocardial infarction, diabetes, and dyslipidemia diagnosed by professional doctors or receiving treatment for these diseases.

### 2.5. Statistical Analysis

Mean daily dietary intake and standard error (SE) for 11 micronutrients and total energy were estimated by BMI status in each subpopulation. Estimated average requirement (EAR) represents the average daily nutrient intake level that meets the adequacy requirement for half of the healthy individuals in a life stage or gender group [15], and the National Health Commission of People’s Republic of China released the most recent EARs as recommendations for the most micronutrient intakes since 2017 [16,17,18,19]. The prevalence of Chinese adults not meeting the EAR was further estimated for selected micronutrients in each subpopulation separately by their BMI status. Participants with two or more dietary vitamin intakes below EARs, with two or more dietary mineral intakes below EARs, or with two or more dietary micronutrients intakes below EARs were defined as multiple vitamin deficiencies, multiple mineral deficiencies, or multiple micronutrient deficiencies, respectively. For the characteristics of the participants, categorical variables were expressed as frequencies and percentages and compared by using chi-square analysis; means and SEs were used to describe the distribution of continuous variables and examined by using generalized linear regression models. In addition, generalized linear regression models were used to evaluate whether the mean dietary intake and the dietary density intake of micronutrient differs by gender in the whole study population and whether the intake and the density intake differs by BMI status in every individual subpopulation through the incorporation of covariates (i.e., age, BMI status) into the models.

We conducted all statistical analyses using SAS version 9.4 (SAS Institute, Inc., Cary, NC, USA). All statistical tests were two-tailed and considered significant at *p* < 0.05.

## 3. Results

### 3.1. Participant Characteristics

Of the participants, 56.03% were men and 43.97% were women. More than half of the subjects were 45–59 years old (54.32%); 85.58% of the subjects had primary or above education level (Table 1). Less than 60% of participants were of normal weight. Lower blood pressure and lower prevalence of diet-related chronic diseases history were observed in underweight and normal-weight men and women in comparison with those who were overweight or obese (data not shown).

### 3.2. Double Burden of Undernutrition and Overnutrition

Among the studied population, the prevalence of vitamin or mineral deficiencies was as high as 96.80% or 99.97%, respectively. Nearly half of subjects faced the problem of DBM (43.55%), of which 39.58% of adults experienced overweight/obesity and micronutrient deficiency simultaneously, along with 3.97% of adults affected by underweight and micronutrient deficiency concurrently. The prevalence of malnutrition in all forms was higher in men, the elder age group, medium yearly income level group, current drinking group, and those with history of diet-related chronic diseases, which was consistent with the distribution of overweight/obesity and micronutrient deficiency (Table 1).

### 3.3. Disparity in Micronutrient Intake across BMI Levels

The dietary intakes of the following micronutrients of men were significantly higher than those of women across BMI categories: thiamin, riboflavin, phosphorus, magnesium, and zinc (Table 2). Compared with normal-weight, overweight, and obese men, those counter women had significantly higher dietary density intakes of riboflavin, vitamin C, and calcium. Normal-weight women also had higher dietary density intakes of magnesium and copper, obese women had higher dietary density intakes of thiamin and iron, while overweight women had lower dietary density intakes of zinc and selenium, compared with those counterparts (Table 3).

### 3.4. Disparity in Micronutrient Deficiency Status across BMI Levels

**Figure 1:**
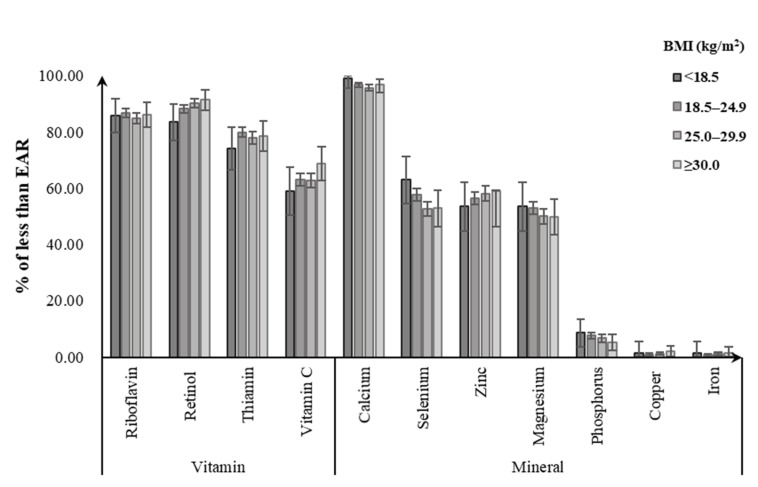
Percentage of Chinese men aged 18–59 years with dietary micronutrient intakes below the estimated average requirements (EARs) by body weight status.

**Figure 2:**
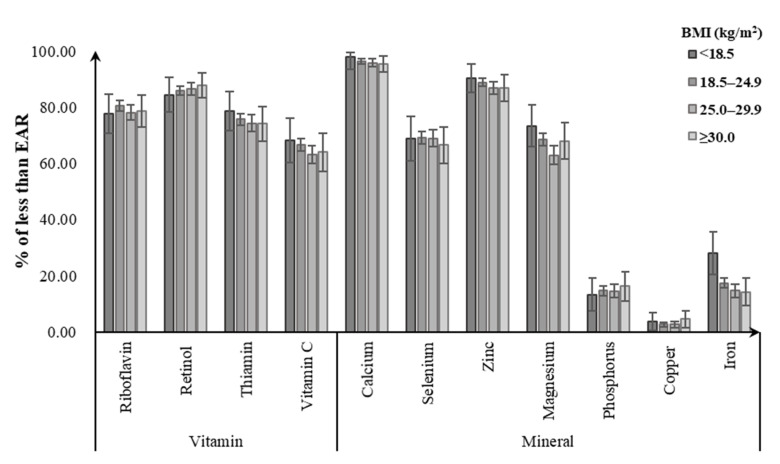
Percentage of Chinese women aged 18–59 years with dietary micronutrient intakes below the estimated average requirements (EARs) by body weight status.

Of men, 85.8–86.3% did not meet the EAR for riboflavin, with the noticeably highest prevalence for the obese group. Likewise, obese men did not meet the EAR for dietary intakes of retinol (91.4%), vitamin C (67.7%), and zinc (58.8%) from foods, with a greater percentage than for other BMI groups. For dietary calcium intake, close to 100% of men failed to meet the EAR regardless of their BMI status. Meanwhile, 74.1–79.9% of men were not meeting the EARs for dietary intake of thiamin, with the highest prevalence for the normal-weight group. Underweight men had the highest prevalence of not meeting EAR for selenium (62.9%), magnesium (53.3%), and phosphorus (8.7%), followed by normal-weight (selenium: 57.7%; magnesium: 53.1%; phosphorus: 7.6%). Less than 3% of men in all BMI groups consumed copper and iron intakes from foods below the EARs (Figure 1). Among women, the percentage having a intake below the EAR tended to be higher in the underweight group for calcium (97.8%), zinc (90.1%), thiamin (78.5%), magnesium (73.3%), vitamin C (68.2%), and iron (28.2%), followed by the normal-weight group (calcium: 96.4%, zinc: 88.9%, thiamin: 75.7%, magnesium: 68.5%, vitamin C: 66.7%, iron: 17.4%). Obese women had the highest prevalence of not meeting the EAR for retinol (87.8%), phosphorus (16.2%), and copper (4.6%). Similarly, close to 100% of women had dietary calcium intake below the EAR in all BMI groups (Figure 2).

**Figure 3:**
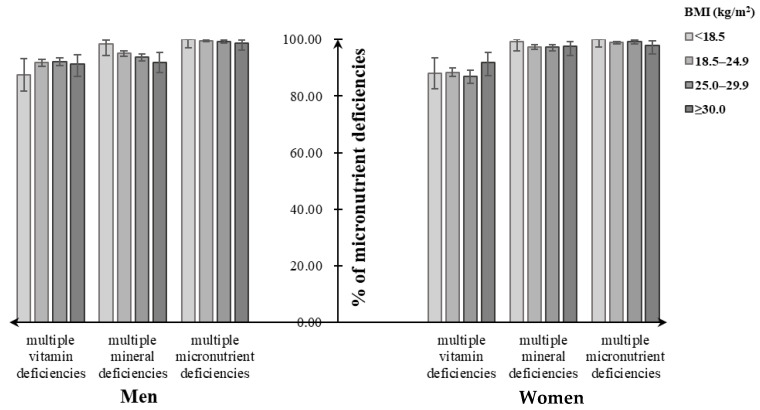
Percentage of multiple micronutrient deficiencies among Chinese adults aged 18–59 years by their body weight status.

**Figure 4:**
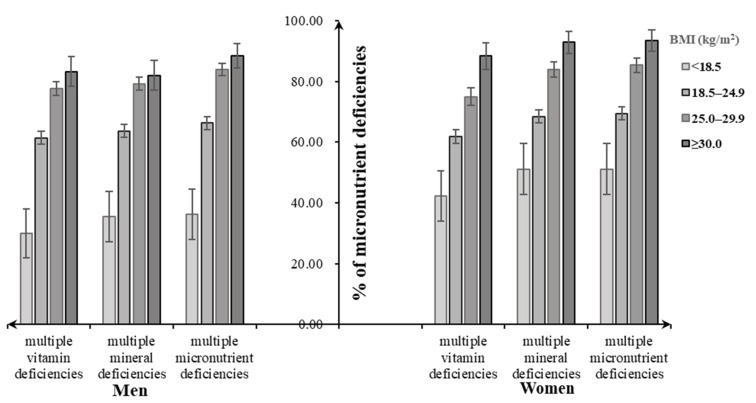
Percentage of malnutrition forms including multiple micronutrient deficiencies and diet-related chronic diseases among Chinese adults aged 18–59 years by body weight status.

Assessment of dietary micronutrient intakes showed that close to 100% of participants had multiple micronutrient deficiencies, not meeting the EARs for at least two dietary selected micronutrient intakes, regardless of their BMI categories. Similarly, close to 100% of underweight men and women had multiple mineral deficiencies (men: 98.4%, women: 99.3%), followed by normal-weight (men: 95.1%, women: 97.5%), overweight (men: 93.8%, women: 97.4%), and obese individuals (men: 91.8%, women: 97.2%). For multiple vitamin deficiencies, the overweight men tended to have a higher percentage (92.2%), while the overweight women had a lower one (86.9%) (Figure 3). Both men and women suffering from diet-related chronic diseases had increasing percentages of multiple micronutrient deficiencies and its subtypes with the increasing BMI status, which were lower than that without consideration of chronic diseases (Figure 4).

## 4. Discussion

Using CHNS 2015, this study quantified the DBM among Chinese adults aged 18 to 59 years as well as examined whether daily dietary micronutrient status varies by BMI categories. Our results indicated that nearly half of subjects faced the problem of DBM, of which 40% of adults experienced overweight/obesity and micronutrient deficiency simultaneously; more than 80% of participants had at least two selected vitamin or mineral deficiencies in all BMI categories, and there were weight composition disparities in dietary intake status for several key micronutrients.

The papers in 2019 *Lancet* Series [1,20,21,22] which highlighted that there are multiple forms of malnutrition that overlap in different ways and in different places were timely to take the issue of malnutrition in all forms or the DBM a step further. Coexistence of multiple forms of malnutrition is a growing public health problem in many places, especially in low- and middle-income countries. In rural Kenya, 19% of the adults in the sample (mean age: 46.2 ± 12.9 years) were affected by overweight/obesity and micronutrient deficiency simultaneously in 2016 [23]. In India, the prevalence of both underweight and overweight/obesity was excessing 20% in 2015–2016, which almost doubled from 2005–2006 to 2015–2016 [24]. Among reproductive age (15–49 years) Vietnamese women, 20.5% of the population were underweight, 20.0% were overweight, including only 2.4% of obese women, and more than 60% of women were affected by the micronutrient deficiency [25]. Among our studied population, 44% of Chinese adults experienced the DBM in 2015, of which 40% of adults were affected by overweight/obesity and micronutrient deficiency simultaneously, which was more prevalent than in the above studies. Mineral insufficiency (99.97%) was the highest form of malnutrition among Chinese adults, followed by vitamin deficiency (96.80%), overweight (33.07%), obesity (6.51%), and underweight (3.97%). In China, the DBM phenomenon was a burning issue for the Sustainable Development Goal of ending malnutrition in all its forms by 2030.

Daily consumptions of dietary micronutrients were relatively low among Chinese men and women because a large number of individuals did not reach the Chinese EAR for several key micronutrient intakes from foods. Mean daily dietary intakes of vitamins among men and women from all BMI categories in our study were lower than that in Korea [26], while the inverse correlation was found for most mineral intakes. Studies in other countries reported that lower intakes of vitamins and minerals were observed across higher BMI categories [2,3], which indicates that BMI is inversely associated with micronutrient intakes and obesity is associated with chronic inadequate intakes of micronutrients [27]. Consistently, the current study observed that the prevalence of insufficiencies for riboflavin, retinol, vitamin C, and zinc from foods was particularly high among obese men; meanwhile, obese women did not meet the Chinese EAR for dietary intakes of retinol, phosphorus, and copper from food, with a greater percentage than for other BMI groups. However, the similar correlation between weight status and the dietary micronutrient intake was not observed among male and female participants in this study. A trend towards a decrease in most micronutrient intakes over the study period from 1991 to 2015 [5], and multiple diversities in age, study population, the study design, and dietary structures, may make these differences.

Dietary calcium is one of the essential micronutrients in keeping bones healthy [7], and may have beneficial effects on adiposity, insulin resistance, dyslipidemia, and blood pressure [28]. However, dietary calcium inadequacy has been identified as one of the most prevalent deficiencies in the Chinese adult population [7], which was not much different from that in foreign individuals [29]. Of note, nearly all of Chinese men and women aged 18 to 59 years failed to reach the EAR for dietary calcium intake regardless of their BMI status in our study. Actions therefore on improving the population’s poor dietary calcium intake, such as nutritional interventions on increasing dietary intakes of milk and dairy products, are necessary to take. Dietary retinol, another key micronutrient concern, plays an important role in vision, immunity, embryological development, and growth hormone production [30]. A study presented that approximately 87% of Chinese adults consumed less retinol than the EAR, and only 6% of adults consumed more than Chinese recommended nutrient intake in 2015 [6]. Consistently, there was also a substantial percentage of Chinese adults who had low intakes of retinol in all BMI groups in our study. This indicated that dietary retinol insufficiency remained a major micronutrient deficit among Chinese men and women. In addition, we observed dietary zinc inadequacy prevailed among women, and the prevalence was particularly high in the underweight group. Zinc deficit in the diet was associated with adverse long-term effects on growth, immunity, and metabolic status of surviving offspring [31]. There is a need to address possible effective approaches or guidance for dietary diversification and management.

Malnutrition increased the risk of diverse forms of ill health, by imposing interconnected biological pathways, involving imbalance of the gut microbiome, inflammation, metabolic dysregulation, and impaired insulin signaling [20]. Moreover, vitamin and mineral deficiencies have brought great impact on the economic development of almost every developing country, with the direct economic loss accounting for about 3% to 5% of the global gross domestic product [32]. More than 80 percent of participants included in this study had vitamin or mineral insufficiencies regardless of their weight status, leaving that individuals experiencing either undernutrition or overnutrition (overweight/obesity) may also suffer from micronutrient deficiencies, which may be shaped both by food systems that fail to provide all people with healthy, safe, affordable, and sustainable diets, and by suboptimal diets [20,33] for a relatively small percent of Chinese adults reaching the recommendation of Chinese dietary guidelines [34]. Considering both diet and disease at the same time, multiple micronutrient deficits were more prevalent in overweight and obese men and women, which may relate to the high prevalence of chronic diseases in overweight and obese groups, also indicating multiple forms of malnutrition co-exist. Importantly, keeping the adequate dietary micronutrient intake is a key factor in weight control or management because many micronutrients (i.e, retinol, vitamin C, calcium, and zinc) are involved in important metabolic and endocrine processes [35], and the extent to which malnutrition leads to overt noncommunicable diseases depends strongly on subsequent nutritional status [20]. There is a need to focus on effective strategies for double-duty actions that address multiple forms of malnutrition, as discussed in our paper.

One strength of this study is the use of CHNS 2015 data with a large national population-based study sample. Dietary data and anthropometric measurements were collected by trained, standardized, and certified interviewers and combined to offer relatively valid estimates of diets for the sex-specific subpopulations of interest. Our results are useful to assess dietary micronutrient status in Chinese population, which would help reinforce recommendations, interventions, and strategies specific to Chinese men and women. However, it should be noted that there are some limitations in our study. The three consecutive days of 24 h dietary recalls used in CHNS can offer a relatively valid estimate of dietary nutrient intakes, which has been tested [36]; however, it posed the risk of underestimating the dietary intakes for episodically consumed foods. Also, the diet data collected between August and November of each wave may not reflect seasonal differences in micronutrient consumption. As well, recall bias from self-reported diet may exist. Although dietary supplement use was far lower in China, unlike some western counties, it may lead to little bias of the dietary micronutrient intake assessment because of the limited information on the use of supplement and medication in CHNS. In addition, the potential reverse causation resulting from the cross-sectional nature of our study is a particular concern when assessing associations between dietary status and body weight, because dietary behavior is influenced by individuals’ perception of their weight status. Nonetheless, these findings provide an important assessment of dietary micronutrient intakes among Chinese men and women aged 18 to 59 years, as well as by different BMI status.

Previous study recommended the use of lower cutoffs for defining the overweight and obesity (overweight: BMI ≤ 24.0 kg/m^2^; obesity: BMI ≤ 28.0 kg/m^2^) in Chinese than those used for whites [37], given lower BMI cutoffs were found in Chinese corresponding to whites for having the same fat and fat distribution. We provided the supplement tables and figures to clarify the DBM phenomenon with the cutoff values of Asian population further (Figures S1–S4 and Table S1). Although the small variance of each micronutrient deficiency across overweight and obese groups defined by the cutoff values of WHO and Asian was observed, 53% of Chinese adults were affected by DBM and half of them experienced overweight/obesity and micronutrient deficiency simultaneously, which introduces the heavy situation of DBM among Chinese in the domestic aspect and also clarifies individuals experiencing over-nutrition (overweight/obesity) were also suffering from micronutrient deficiencies.

## 5. Conclusions

The present study showed that Chinese adults aged 18–59 years faced the burning issue of DBM; Chinese men and women across different BMI categories had a poor-quality diet, with intakes well below the EARs for several key micronutrients (i.e., calcium, retinol, riboflavin, thiamin, vitamin C, selenium, zinc, and magnesium), and these inadequacies were more prevalent in underweight and obese groups. The study also showed the extent to which multiple micronutrient deficiencies overlap in these settings. Understanding and controlling micronutrient inadequacies for Chinese adults can have significant public health impact. Therefore, the promotion of population-wide intakes of more nutrient-dense foods—through encouragement of self-selection in food supply and reinforcement of precise dietary supplement programs or interventions—is needed to tackle the inadequate intake of micronutrients for target subgroups in China.

## Figures and Tables

**Table 1:** Characteristics of study participants and prevalence of multiple forms of malnutrition, China Health and Nutrition Survey (CHNS) 2015 ^1^.

Characteristics	Participants	Underweight	Overweight	Obesity	Vitamin Deficiency	Mineral Deficiency	Underweight and Micronutrient Deficiency	Overweight/Obesity and Micronutrient Deficiency	Malnutrition ^2^
Number of subjects	6602(100.00)	262(3.97)	2183(33.07)	430(6.51)	6391(96.80)	6600(99.97)	262(3.97)	2613(39.58)	2875(43.55)
Sex									
Men	3699(56.03)	127(3.43) ^3^	1345(36.36) ^3^	233(6.30)	3610(97.59) ^3^	3697(99.95)	127(3.43)	1578(42.66) ^3^	1705(46.09) ^3^
Women	2903(43.97)	135(4.65)	838(28.87)	197(6.79)	2781(95.80)	2903(100.00)	135(4.65)	1035(35.65)	1170(40.30)
Age									
18–44 years	3016(45.68)	185(6.13) ^3^	887(29.41) ^3^	188(6.23)	2928(97.08)	3016(100.00)	185(6.13) ^3^	1075(35.64) ^3^	1260(41.78) ^3^
45–59 years	3586(54.32)	77(2.15)	1296(36.14)	242(6.75)	3463(96.57)	3584(99.94)	77(2.15)	1538(42.89)	1615(45.04)
Education									
Illiterate	952(14.42)	40(4.20)	329(34.56)	66(6.93)	929(97.58)	952(100.00)	40(4.20)	395(41.49) ^3^	435(45.69)
Primary school	2572(38.96)	88(3.42)	878(34.14)	179(6.96)	2498(97.12)	2571(99.96)	88(3.42)	1057(41.10)	1145(44.52)
≥High school	3078(46.62)	134(4.35)	976(31.71)	185(6.01)	2964(96.30)	3077(99.97)	134(4.35)	1161(37.72)	1295(42.07)
Urbanicity index									
Low	2208(33.44)	93(4.21)	712(32.25)	139(6.30) ^3^	2155(97.60) ^3^	2207(99.95)	93(4.21)	851(38.54)	944(42.75)
Medium	2197(33.28)	92(4.19)	710(32.32)	166(7.56)	2124(96.68)	2197(100.00)	92(4.19)	876(39.87)	968(44.06)
High	2197(33.28)	77(3.50)	761(34.64)	125(5.69)	2112(96.13)	2196(99.95)	77(3.50)	886(40.33)	963(43.83)
Yearly Income									
Low	2199(33.31)	105(4.77)	685(31.15) ^3^	142(6.46)	2141(97.36)	2197(99.91)	105(4.77)	827(37.61) ^3^	932(42.38) ^3^
Medium	2202(33.35)	80(3.63)	774(35.15)	157(7.13)	2129(96.68)	2202(100.00)	80(3.63)	931(42.28)	1011(45.91)
High	2201(33.34)	77(3.50)	724(32.89)	131(5.95)	2121(96.37)	2201(100.00)	77(3.50)	855(38.84)	932(42.34)
Physical activity									
Low	2200(33.32)	96(4.36)	721(32.77)	158(7.18)	2123(96.50)	2199(99.95)	96(4.36)	879(39.95)	975(44.31)
Medium	2201(33.34)	84(3.82)	707(32.12)	138(6.27)	2125(96.55)	2201(100.00)	84(3.82)	845(38.39)	929(42.21)
High	2201(33.34)	82(3.73)	755(34.30)	134(6.09)	2143(97.36)	2200(99.95)	82(3.73)	889(40.39)	971(44.12)
Current smoking									
No	4516(68.40)	185(4.10)	1454(32.20) ^3^	305(6.75)	4360(96.55)	4515(99.98)	185(4.10)	1759(38.95)	1944(43.05)
Yes	2086(31.60)	77(3.69)	729(34.95)	125(5.99)	2031(97.36)	2085(99.95)	77(3.69)	854(40.94)	931(44.63)
Current drinking									
No	4306(65.22)	197(4.58) ^3^	1340(31.12) ^3^	285(6.62)	4150(96.38) ^3^	4306(100.00) ^3^	197(4.58) ^3^	1625(37.73) ^3^	1822(42.31) ^3^
Yes	2296(34.78)	65(2.83)	843(36.72)	145(6.32)	2241(97.60)	2294(99.91)	65(2.83)	988(43.03)	1053(45.86)
History of diet-related chronic diseases									
No	1692(25.63)	147(8.69) ^3^	327(19.33)	34(2.01)	1649(97.46)	1691(99.94)	147(8.69) ^3^	361(21.33) ^3^	508(30.02) ^3^
Yes	4910(74.37)	115(2.34)	1856(37.80)	396(8.07)	4742(96.58)	4909(99.98)	115(2.34)	2252(45.87)	2367(48.21)

^1^ All values are n (%). CHNS, China Health and Nutrition Survey; ^2^ malnutrition defined as having underweight/overweight/obesity and micronutrient deficiency; ^3^ showed a significant difference in the prevalence of multiple forms of malnutrition tested by Chi-square test (*p* < 0.05).

**Table 2:** Estimated daily micronutrient intakes from foods by category of body mass index (BMI) in men and women aged 18–59 years, CHNS 2015 (N = 6602) ^1^.

Micronutrient	Men				Women			
	BMI < 18.5 kg/m^2^	BMI 18.5–24.9kg/m^2^	BMI 25.0–29.9 kg/m^2^	BMI ≥ 30.0 kg/m^2^	BMI <18.5 kg/m^2^	BMI 18.5–24.9 kg/m^2^	BMI 25.0–29.9 kg/m^2^	BMI ≥ 30.0 kg/m^2^
Retinol (μg RAE)	337.53 ± 71.48	373.40 ± 17.97	358.47 ± 21.90	346.98 ± 52.58	376.94 ± 53.60	332.48 ± 14.68	354.89 ± 21.27	294.52 ± 43.55
Thiamin (mg) ^2^	0.96 ± 0.04	0.93 ± 0.01	0.96 ± 0.01	0.94 ± 0.03	0.82 ± 0.03 ^4^	0.82 ± 0.01 ^4^	0.83 ± 0.01 ^4^	0.82 ± 0.03 ^4^
Riboflavin (mg) ^2^	0.87 ± 0.03	0.84 ± 0.01	0.88 ± 0.01	0.86 ± 0.02	0.77 ± 0.03 ^4^	0.77 ± 0.01 ^4^	0.79 ± 0.01 ^4^	0.78 ± 0.03 ^4^
Vitamin C (mg)	81.76 ± 9.84	81.13 ± 2.47	88.23 ± 3.02	73.24 ± 7.24	79.77 ± 14.79	87.54 ± 4.05	91.13 ± 5.87	93.52 ± 12.02
Calcium (mg)	382.74 ± 18.88	384.44 ± 4.75	400.13 ± 5.78	383.28 ± 13.89	354.73 ± 18.86	368.30 ± 5.16 ^4^	369.05 ± 7.48 ^4^	373.34 ± 15.32
Phosphorus (mg) ^3^	987.79 ± 30.08	1012.60 ± 7.56	1054.06 ± 9.22	1054.13 ± 22.13	880.56 ± 29.92 ^4^	895.98 ± 8.19 ^4^	913.99 ± 11.87 ^4^	889.60 ± 24.31 ^4^
Magnesium (mg) ^3^	285.88 ± 10.12	286.94 ± 2.54	301.59 ± 3.10	304.43 ± 7.45	251.43 ± 11.24 ^4^	261.81 ± 3.08 ^4^	272.53 ± 4.46 ^4^	264.51 ± 9.13 ^4^
Iron (mg) ^3^	23.64 ± 1.02	23.86 ± 0.26	24.79 ± 0.31	24.52 ± 0.75	20.30 ± 1.03 ^4^	21.19 ± 0.28 ^4^	21.49 ± 0.41 ^4^	22.05 ± 0.84
Zinc (mg)	11.45 ± 0.37	11.54 ± 0.09	11.89 ± 0.11	11.78 ± 0.27	9.94 ± 0.36 ^4^	10.04 ± 0.10 ^4^	10.01 ± 0.14 ^4^	9.82 ± 0.29 ^4^
Copper (mg) ^3^	1.71 ± 0.08	1.81 ± 0.02	1.89 ± 0.03	1.84 ± 0.06	1.62 ± 0.08	1.66 ± 0.02 ^4^	1.68 ± 0.03 ^4^	1.58 ± 0.06 ^4^
Selenium (μg) ^3^	46.76 ± 2.58	51.88 ± 0.65	54.35 ± 0.79	53.79 ± 1.90	44.63 ± 2.19	45.22 ± 0.60 ^4^	44.67 ± 0.87 ^4^	44.66 ± 1.78 ^4^

^1^ All values are means ± SEs. CHNS, China Health and Nutrition Survey; RAE, retinol activity equivalents. ^2^ *p* < 0.05 across BMI categories for women calculated by using generalized linear models with adjustment for age. ^3^ *p* < 0.05 across BMI categories for men calculated by using generalized linear models with adjustment for age. ^4^ Significantly different from men within BMI category calculated by using generalized linear models with adjustment for age, *p* < 0.05.

**Table 3:** Estimated daily micronutrient density intake from foods by category of BMI in men and women aged 18–59 years, CHNS 2015 (N = 6602) ^1^.

Micronutrient	Men				Women			
	BMI < 18.5 kg/m^2^	BMI 18.5–24.9kg/m^2^	BMI 25.0–29.9 kg/m^2^	BMI ≥ 30.0 kg/m^2^	BMI < 18.5 kg/m^2^	BMI 18.5–24.9 kg/m^2^	BMI 25.0–29.9 kg/m^2^	BMI ≥ 30.0 kg/m^2^
Retinol (μg RAE/1000 kcal)	152.64 ± 31.63	179.15 ± 7.95	163.23 ± 9.69	152.12 ± 23.27	199.02 ± 27.22	183.17 ± 7.45	184.88 ± 10.80	168.97 ± 22.11
Thiamin (mg/1000 kcal)	0.43 ± 0.01	0.43 ± 0.00	0.43 ± 0.00	0.41 ± 0.01	0.43 ± 0.01	0.43 ± 0.01	0.43 ± 0.01	0.44 ± 0.01 ^2^
Riboflavin (mg/1000 kcal)	0.40 ± 0.01	0.40 ± 0.00	0.40 ± 0.00	0.38 ± 0.01	0.41 ± 0.01	0.42 ± 0.01 ^2^	0.42 ± 0.01 ^2^	0.42 ± 0.01 ^2^
Vitamin C (mg/1000 kcal)	38.32 ± 4.12	39.37 ± 1.04	40.14 ± 1.26	33.93 ± 3.03	42.28 ± 7.17	47.49 ± 1.96 ^2^	48.55 ± 2.84 ^2^	53.76 ± 5.82 ^2^
Calcium (mg/1000 kcal)	183.26 ± 8.10	184.18 ± 2.04	183.24 ± 2.48	175.35 ± 5.96	194.49 ± 9.32	201.15 ± 2.55 ^2^	197.58 ± 3.70 ^2^	207.27 ± 7.57 ^2^
Phosphorus (mg/1000 kcal)	454.70 ± 9.05	476.52 ± 2.27	477.47 ± 2.77	471.39 ± 6.65	468.42 ± 9.56	481.41 ± 2.62	479.55 ± 3.79	483.24 ± 7.77
Magnesium (mg/1000 kcal)	131.19 ± 3.37	135.05 ± 0.85	136.24 ± 1.03	135.97 ± 2.48	132.43 ± 3.60	139.35 ± 0.99^2^	141.61 ± 1.43 ^2^	143.15 ± 2.93
Iron (mg/1000 kcal)	10.95 ± 0.40	11.22 ± 0.10	11.20 ± 0.12	10.92 ± 0.29	10.71 ± 0.39	11.27 ± 0.11	11.11 ± 0.16	11.82 ± 0.32 ^2^
Zinc (mg/1000 kcal)	5.25 ± 0.12	5.42 ± 0.03	5.37 ± 0.04	5.23 ± 0.09	5.24 ± 0.12	5.35 ± 0.03	5.24 ± 0.05 ^2^	5.35 ± 0.10
Copper (mg/1000 kcal)	0.78 ± 0.03	0.85 ± 0.01	0.84 ± 0.01	0.82 ± 0.02	0.86 ± 0.03 ^2^	0.88 ± 0.01 ^2^	0.87 ± 0.01	0.85 ± 0.02
Selenium (μg/1000 kcal)	21.34 ± 1.21	24.59 ± 0.30	24.69 ± 0.37	23.78 ± 0.89	23.90 ± 1.01	24.21 ± 0.28	23.37 ± 0.40 ^2^	24.20 ± 0.82

^1^ All values are means ± SEs. CHNS, China Health and Nutrition Survey; RAE, retinol activity equivalents. ^2^ Significantly different from men within BMI category calculated by using generalized linear models with adjustment for age, *p* < 0.05.

## Supplementary Materials

The following are available online at https://www.mdpi.com/2072-6643/12/9/2811/s1, Figure S1. Percentage of Chinese men aged 18–59 years with dietary micronutrient intakes below the Estimated Average Requirements (EARs) by body weight status; Figure S2. Percentage of Chinese women aged 18–59 years with dietary micronutrient intakes below the Estimated Average Requirements (EARs) by body weight status; Figure S3. Percentage of multiple micronutrient deficiencies among Chinese adults aged 18–59 years by their body weight status; Figure S4. Percentage of malnutrition forms including multiple micronutrient deficiencies and diet-related chronic diseases among Chinese adults aged 18–59 years by body weight status; Table S1. Characteristics of study participants and prevalence of multiple forms of malnutrition, CHNS 2015 ^1^.
