# Supplementary material for: Intra-Individual Double Burden of Malnutrition among Adults in China: Evidence from the China Health and Nutrition Survey 2015
Source: Nutrients. 2020 Sep 14;12(9):2811. doi: 10.3390/nu12092811 (PMC7551383; doi:10.3390/nu12092811)
Supplement: Supplementary file 1 [file nutrients-12-02811-s001.zip › nutrients-911714-supplementary.docx]

Figure S1. Percentage of Chinese men aged 18–59 years with dietary micronutrient intakes below the Estimated Average Requirements (EARs) by body weight status.

Figure S2. Percentage of Chinese women aged 18–59 years with dietary micronutrient intakes below the Estimated Average Requirements (EARs) by body weight status.

Women

Figure S3. Percentage of multiple micronutrient deficiencies among Chinese adults aged 18–59 years by their body weight status.

Women

Figure S4. Percentage of malnutrition forms including multiple micronutrient deficiencies and diet-related chronic diseases among Chinese adults aged 18–59 years by body weight status.

| Table S1. Characteristics of study participants and prevalence of multiple forms of malnutrition, CHNS 2015 ^1^ | | | | | |
| --- | --- | --- | --- | --- | --- |
| **Characteristics** | Overweight | Obesity | Underweight and micronutrient deficiency | Overweight/obesity and micronutrient deficiency | Malnutrition^2^ |
| Number of subjects | 2312(35.02) | 990(15.00) | 262(3.97) | 3302(50.02) | 3564(53.98) |
| Sex |  |  |  |  |  |
| Men | 1383(37.39) ^3^ | 578(15.63) | 127(3.43) ^3^ | 1961(53.01) ^3^ | 2088(56.45) ^3^ |
| Women | 929(32.00) | 412(14.19) | 135(4.65) | 1341(46.19) | 1476(50.84) |
| Age |  |  |  |  |  |
| 18-44 years | 933(30.94) ^3^ | 414(13.73) ^3^ | 185(6.13) ^3^ | 1347(44.66) ^3^ | 1532(50.80) ^3^ |
| 45-59 years | 1379(38.46) | 576(16.06) | 77(2.15) | 1955(54.52) | 2032(56.66) |
| Education |  |  |  |  |  |
| Illiterate | 340(35.71) | 148(15.55) | 40(4.20) | 488(51.26) | 528(55.46) |
| Primary school | 901(35.03) | 404(15.71) | 88(3.42) | 1305(50.74) | 1393(54.16) |
| ≥High school | 1071(34.80) | 438(14.23) | 134(4.35) | 1509(49.03) | 1643(53.38) |
| Urbanicity index | |  |  |  |  |
| Low | 732(33.15) | 331(14.99) | 93(4.21) | 1063(48.14) | 1156(52.36) |
| Medium | 780(35.50) | 333(15.16) | 92(4.19) | 1113(50.66) | 1205(54.85) |
| High | 800(36.41) | 326(14.84) | 77(3.50) | 1126(51.25) | 1203(54.76) |
| Yearly Income |  |  |  |  |  |
| Low | 712(32.38) ^3^ | 336(15.28) | 105(4.77) | 1048(47.66) ^3^ | 1153(52.43) |
| Medium | 814(36.97) | 338(15.35) | 80(3.63) | 1152(52.32) | 1232(55.95) |
| High | 786(35.71) | 316(14.36) | 77(3.50) | 1102(50.07) | 1179(53.57) |
| Physical activity | |  |  |  |  |
| Low | 747(33.95) | 358(16.27) | 96(4.36) | 1105(50.23) | 1201(54.59) |
| Medium | 775(35.21) | 299(13.58) ^3^ | 84(3.82) | 1074(48.80) | 1158(52.61) |
| High | 790(35.89) | 333(15.13) | 82(3.73) | 1123(51.02) | 1205(54.75) |
| Current smoking | |  |  |  |  |
| No | 1556(34.46) | 692(15.32) | 185(4.10) | 2248(49.78) | 2433(53.88) |
| Yes | 756(36.24) | 298(14.29) | 77(3.69) | 1054(50.53) | 1131(54.22) |
| Current drinking | |  |  |  |  |
| No | 1461(33.93) ^3^ | 623(14.47) | 197(4.58) ^3^ | 2084(48.40) ^3^ | 2281(52.97) ^3^ |
| Yes | 851(37.06) | 367(15.98) | 65(2.83) | 1218(53.05) | 1283(55.88) |
| History of diet-related chronic diseases | | |  |  |  |
| No | 407(24.05)^3^ | 90(5.32)^3^ | 147(8.69)^3^ | 497(29.37)^3^ | 644(38.06)^3^ |
| Yes | 1905(38.80) | 900(18.33) | 115(2.34) | 2805(57.13) | 2920(59.47) |
| ^1^ All values are n (%). CHNS, China Health and Nutrition Survey;.^2^ Malnutrition defined as having underweight/overweight (24.0≤BMI< 28.0 kg/m^2^ )/obesity (BMI≥28.0 kg/m^2^ ) and micronutrient deficiency; ^3^ Showed a significant difference in the prevalence of multiple forms of malnutrition tested by Chi-square test (p < 0.05). | | | | | |
